# Diffusion Kurtosis Imaging for Assessing the Therapeutic Response of Transcatheter Arterial Chemoembolization in Hepatocellular Carcinoma

**DOI:** 10.7150/jca.32491

**Published:** 2020-02-10

**Authors:** Zhen-Guo Yuan, Zong-Ying Wang, Meng-Ying Xia, Feng-Zhi Li, Yao Li, Zhen Shen, Xi-Zhen Wang

**Affiliations:** 1 Medical Imaging Center of the Affiliated Hospital, Weifang Medical University, Weifang 261053 P. R.China; 2 Shandong Medical Imaging Research Institute Affiliated to Shandong University, Jinan 250021 P. R. China

**Keywords:** diffusion kurtosis imaging, hepatocellular carcinoma, transcatheter arterial chemoembolization

## Abstract

**Objective**: This study aimed to evaluate the therapeutic response of hepatocellular carcinoma (HCC) after transcatheter arterial chemoembolization (TACE) with diffusion kurtosis imaging (DKI).

**Methods**: Forty-three patients with fifty-nine hepatic cancer nodules were recruited for this study. All patients were treated by TACE. Magnetic resonance imaging (MRI) and DKI (b=0, 800, 1,500, 2,000mm^2^/s) were performed before and one month after initiating TACE. Patients were classified as either progressing groups or non-progressing groups. Mean kurtosis (MK), mean diffusion (MD), and apparent diffusion coefficient (ADC) values of the tumor tissue were analyzed.

**Results**: Twenty-three HCCs were classified as progressing groups, and thirty-six HCCs were non-progressing groups. After TACE, the values of MD and ADC in non-progressing groups (1.92±0.36×10^-3^mm^2^/s, 1.36±0.23×10^-3^mm^2^/s) were greater than progressing groups (1.44±0.32× 10^-3^mm^2^/s, 1.10±0.23×10^-3^mm^2^/s), however, the MK values in non-progressing groups (0.47±0.12) were lower than progressing groups (0.72±0.14). The MK values of tumor among non-progressing patients decreased one month after TACE (0.47±0.12) relative to the preoperative values (0.71±0.12) (*P*<0.05). In the non-progressing groups, the MD and ADC values of tumor after TACE (1.92±0.36×10^-3^mm^2^/s, 1.36±0.23×10^-3^mm^2^/s) became higher than their preoperative values (1.44±0.35×10^-3^mm^2^/s, 1.09±0.22×10^-3^mm^2^/s) (*P*<0.05). In the progressing groups, the MK, MD, and ADC values of tumor after TACE remained similar before TACE (*P*>0.05). The sensitivity, specificity, and AUC of the ROC curve for the assessment of HCC progress after TACE by MK (85.2%, 97.5%, and 0.95, respectively) were greater than by ADC (78.6%, 66.5%, and 0.75, respectively) and MD (76.2%, 64.3%, and 0.71, respectively).

**Conclusions**: DKI for assessing the therapeutic response of TACE in HCC shows great promise. MK is more advantageous in the assessment of HCC progress after TACE.

## Introduction

Hepatocellular carcinoma (HCC) is the fifth most common cancer in the world. With the development of western diet, the incidence of the disease is increasing, posing a great threat to the physical and mental health of the patients 1. Most patients with HCC were accompanied by hepatitis, cirrhosis and finally led to the development of liver cancer. Recurrence and metastasis of HCC as two important factors, influencing the prognostic and long-term therapeutic effect of patients 2.

Radiofrequency ablation, resection, and liver transplantation were the traditional treatment methods for HCC, and surgical excision was considered as the preferred treatment for patients with HCC 3. However, when the patients were manifested as advanced liver cancer at the time of diagnosis, surgical resection was not appropriate. In this case, transcatheter arterial chemoembolization (TACE) was an appropriate choice 4. Previous studies showed that TACE is an accepted method, which can improve the prognosis of HCC patients 5. After the successful application of TACE, blood supply of lesions was blocked, leading to tumor necrosis 6. Moreover, potential risks and complications remain inevitable 7. Thus, an accurate evaluated method of HCC after TACE is important to help guide subsequent therapeutic planning in clinical practice.

Functional magnetic resonance imaging was widely used for the evaluation of patients with HCC because of its excellence in the depiction of soft tissue. As a functional magnetic resonance imaging, diffusion weighted imaging (DWI) was widely used in HCC. Study has shown that DWI was no obvious advantage in predicting local HCC recurrence after TACE compared with gadolinium-enhanced MRI 8. In general, DWI is mainly applied to quantify the diffusion of water molecules with Gaussian distribution, which cannot really reflect the lesion information 9. Diffusion kurtosis imaging (DKI) is an emerging method for detecting diffusion of water molecules. In contrast to the free diffusion, due to the differences in structures and functions of tissues cells, such as cell membranes, the diffusion of water molecules *in vivo* often is an abnormal distribution called non-Gaussianity motion. Pathological changes, such as tumor cell proliferation, fresh angiogenesis and tumor cell necrocytosis, can change the microstructure of tumor tissues. DKI is mainly applied to detect this kind of motion of water molecules to reflect the lesion microstructure. Therefore, DKI was sensitive to the detection of the change of tumor biological behavior. Until now, this technology is primarily focused on central nervous system diseases, such as multiple sclerosis, glioma, cerebral infarction, and Parkinson disease 10-13. Recently, DKI was increasingly used in the study on prostate cancer, breast cancer, and kidney cancer 14-16.

In addition, clinical application of DKI in the liver was increasingly prevalent, especially HCC. For example, DKI was used to evaluate the microvascular invasion of HCC 17. However, the study on prognostic evaluation of HCC after TACE is rare. Thus, our study aimed to apply DKI to assess the therapeutic response of TACE in HCC.

## Materials and Methods

### Patients

This study was approved by the local institute review board, and each patient signed the written informed consent. Eighty-eight consecutive patients with HCC proved by pathology were retrospectively selected from a prospective database between January 2017 and March 2018 in my hospital. Among the 88 patients, 64 were male and 24 were female, aged 43 to 82 years old, with an average age of 60.2. However, in the process of following up, three patients had undergone hepatic lobectomy; twenty-five patients were treated by RFA and 17 patients were untreated. Finally, forty-three patients with fifty-nine hepatic cancer nodules satisfied the inclusion standards and fulfilled various examinations, were recruited for this study. All consecutive patients were proven pathologically to HCC and were treated by TACE. Thirty out of the forty-three patients were males, and thirteen patients were females. The patient age ranged from 25 to 77 years old, and the median age was 57.8. Clinical characteristics of the patients and tumors analyzed in this study are summarized in Table 1.

### Imaging Examination

Conventional magnetic resonance imaging (da PHILIPS Achieva, Netherlands 3.0T MRI) and DKI (b=0, 800, 1,500, 2,000 mm^2^/s) were performed. The standard 18-channel mode was employed for the body-phased array coil.

For DKI sequence scanning, a single-shot echo-planar imaging sequence was employed. The settings were as follows: response time (TR) of 3,407ms, echo time (TE) of 77ms, fractional anisotropy (FA) at 90°, layer thickness at 5mm, layer interval of 1.5mm, field of view (FOV) dimension of 375×305mm^2^, and NEX=3. The b values of 0, 800, 1,500, and 2,000s/mm^2^ were selected. Diffusion-sensitive gradient fields in 30 directions were added to each b value.

### Chemoembolization Technique

TACE was performed by two interventional radiologists with 7-10 years of clinical experience, correspondingly 18. Under diagnostic digital subtraction angiography (DSA) guidance (LCV plus; GE Medical Systems, Milwaukee, Wisconsin), accessing to the techniques of Seldinger, a sheath introducer was placed in the right common femoral artery; a 5 French (F) angiographic catheter (Terumo, Fujinomiya, Japan) was advanced into the common hepatic artery; and a 2.2~2.4 F coaxial catheter (Prograte; Trumo; Medical, Somerset, NJ) was advanced over 0.0016-inch guide wire (Glidewire; Terumo Medical, Somerset, NJ) into the desired hepatic arterial branch. After catheterization, initially, 1000~1500mg of 5-fluorour acil and 30~40mg of hydroxycamptothecine infused into the tumor feeder vessels. Then, an emulsion of 40~50mg Adriamycin and 3~20mL of iodized oil (Lipiodol Ultra Fluid, LaboratoireGuerbet, Aulnay-Sous-Bois, France) was injected through the catheter. Finally, gelatin sponge particles (with a diameter of 1mm, 20~60 particles, Sponjel; Asteras, Tokyo) were administered into the feeder vessels. The dose of iodized oil and Adriamycin depended on the size of tumor and the liver function of patient. The chemoembolization procedure should be stopped, when the tumor stain disappeared or decreased markedly.

### Following up

Each subject was performed contrast-enhanced MRI and DKI after initiating TACE one month, and then followed-up every 3 months. “Technique effectiveness” should be defined as a prospectively defined time point, usually 1-3 months after a treatment cycle, at which point response is assessed at imaging follow-up 19. In this study, we selected one month, which was considered the earliest time point to assess the tumors, may guide timely decision-making for subsequent therapies.

**Figure 1:**
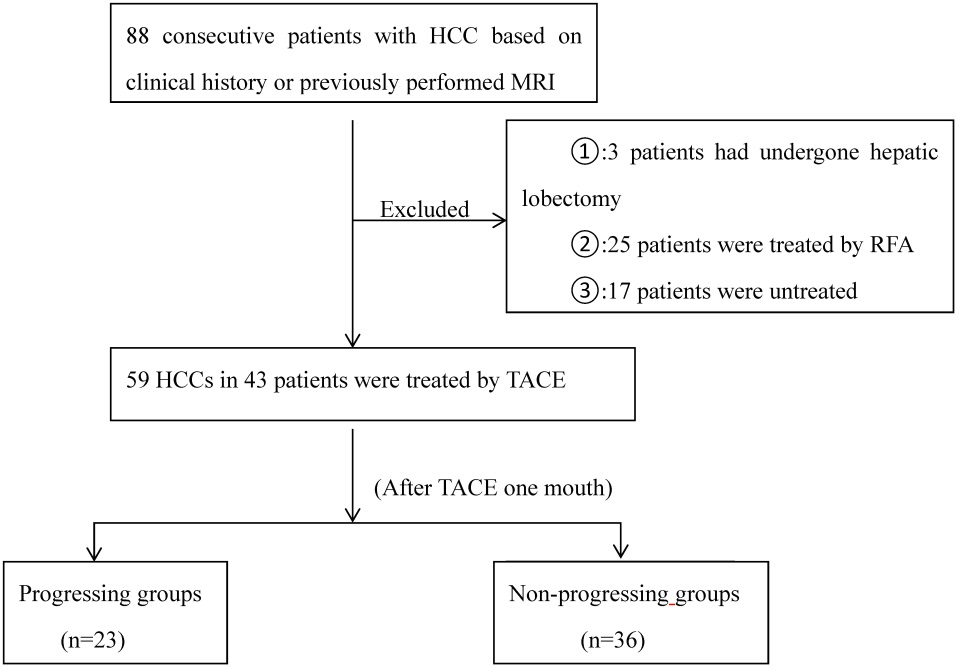
The follow-up flow chart of HCCs

The diameter of HCC nodules we measured was equal to 1cm or greater. According to tumor response, the HCCs were be classified as either progressing groups or non-progressing groups, which was assessed according to the overall mRECIST 20. Non-progressing groups were classified as complete necrosis, partial necrosis and stable nodules. Progressing groups were defined as the sum of the longest diameters of the target tumors increased greater than 20%, or the emergence of one or several liver enhanced nontarget lesions, or new lesions after TACE. Progressing groups were also classified according to metastasis: local recurrence and distant recurrence. Figure 1 represents the flow chart of HCCs.

### Imaging analysis

DKI image post-processing software was provided by PHILIPS. The post-processing was based on the DKI model. According to the DKI theories: S=S0·exp (-b D + b2·D2·K/6), where K is the mean kurtosis (MK), and the mean diffusion (MD) value is similar to the corrected average apparent diffusion coefficient (ADC) value. The raw images from DKI sequence scanning were fed into the post-processing software (DKE). Finally, the MD and MK values were obtained. According to the DWI imaging, when b=0 and b are not equal to 0 (800, 1500, 2000, s/mm^2^), the ADC value was calculated using the formula Sb/S0=exp (-b·ADC). Measurements of the parameters were performed thrice, and MD, MK, and ADC of the three measurements were recorded. The region of interest (ROI) was then selected. The scope of the lesion should be as large as possible. To reduce the error, for each case, lesion ROIs were set at three different positions. The selected range was kept as consistent as possible for each patient. The ROI area of the lesions ranged from 1.0cm^2^ to 2.0cm^2^. The difference in the corresponding parameters and the correlation to the HCC prognosis was analyzed.

### Statistical analysis

Related parameters of DKI, and ADC were subjected to statistical analysis treatments in SPSS 20.0 statistics software. Quantitative data are expressed in the format of means±standard deviations. Analysis of variance (ANOVA) was performed in all parameters and recurrence. When the *P*<0.05, the difference was considered statistically significant. MK, MD, and ADC values of the tumor tissue before and one month after TACE were analyzed using Mann-Whitney tests in both the progressing and non-progressing groups. The efficacy of ADC, MD, and MK was evaluated by the ROC curve. The sensitivity, specificity, and AUC under the ROC curve for the evaluation of HCC viability were also calculated.

## Result

**Figure 2:**
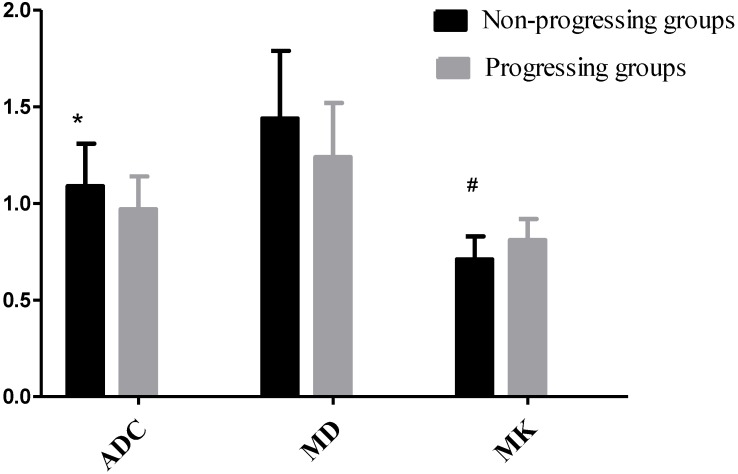
*P<0.05, the non-progressing groups versus progressing groups in ADC before TACE; ^#^*P*<0.05, the non-progressing groups versus progressing groups in MK before TACE.

**Figure 3:**
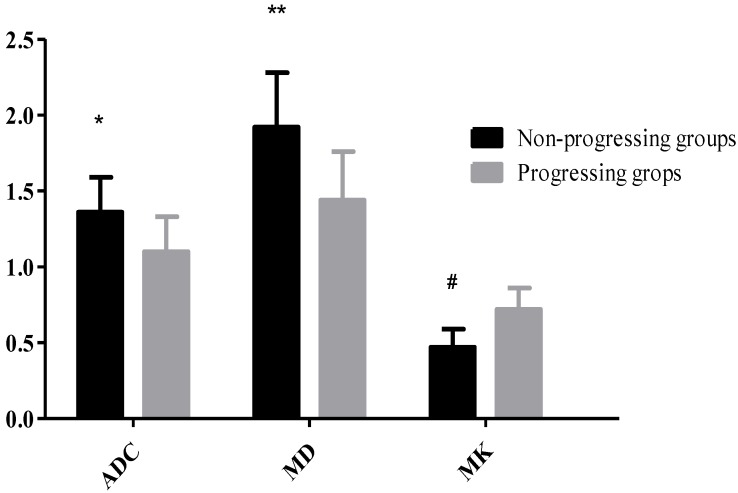
*P<0.05, the non-progressing groups versus progressing groups in ADC after TACE; **P<0.05, the non-progressing groups versus progressing groups in MD after TACE; ^#^*P*<0.05, the non-progressing groups versus progressing groups in MK after TACE.

**Figure 4:**
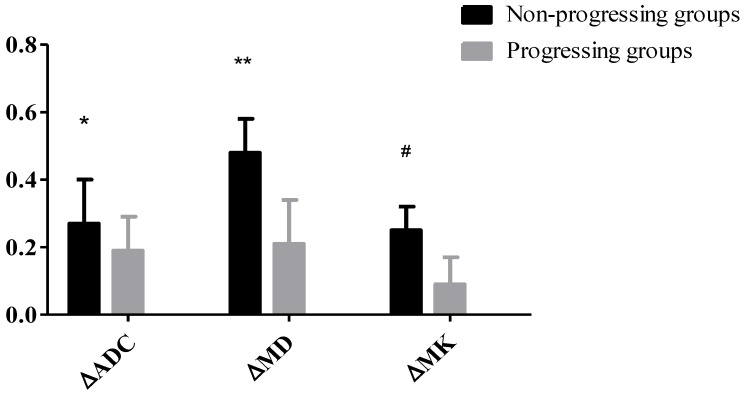
*P<0.05, the non-progressing groups versus progressing groups in ∆ADC after TACE; **P<0.05, the non-progressing groups versus progressing groups in ∆MD after TACE; ^#^*P*<0.05, the non-progressing groups versus progressing groups in ∆MK after TACE.

23 HCC nodules were classified as progressing groups, and 36 HCC nodules were non-progressing groups. Among the 36 HCC nodules, 25 HCCs were complete necrosis, 8 HCC nodules were partial necrosis and 3 HCCs were stable disease. In our study, according to Fig. 2, no significant difference was observed in the preoperative MD values of tumor between the progressing groups and non-progressing groups. However, significant difference was found in the preoperative MK and ADC values of tumor between the progressing groups and non-progressing groups. After TACE, a significant difference was noted in the MK, MD, and ADC values of tumor between the progressing groups and non-progressing groups. After TACE, the values of MD and ADC in non-progressing groups (1.92±0.36×10^-3^mm^2^/s, 1.36± 0.23×10^-3^mm^2^/s) were greater than progressing groups (1.44±0.32×10^-3^mm^2^/s, 1.10±0.23×10^-3^mm^2^/s), however, the MK values in non-progressing groups (0.47±0.12) were lower than progressing groups (0.72±0.14) (Fig. 3). The decrease of the MK values (∆MK) in the non-progressing groups (0.25±0.07) was significantly greater than that observed in the progressing groups (0.09±0.08) (*P*<0.05). Moreover, the increase of the MD and ADC values (∆MD, ∆ADC) in the non-progressing groups (0.48± 0.10×10^-3^mm^2^/s, 0.27±0.13×10^-3^mm^2^/s) was more significant than that observed in the progressing groups (0.21±0.13×10^-3^mm^2^/s, 0.09±0.08×10^-3^mm^2^/s) (*P*<0.05) (Fig. 4).

**Figure 5:**
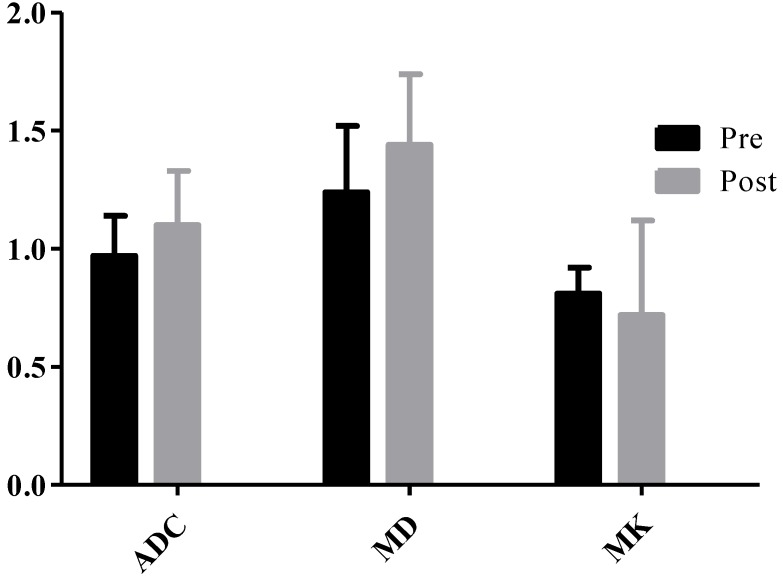
The MK, MD, and ADC values of tumor remained similar before and one month after TACE in the progressing groups.

**Figure 6:**
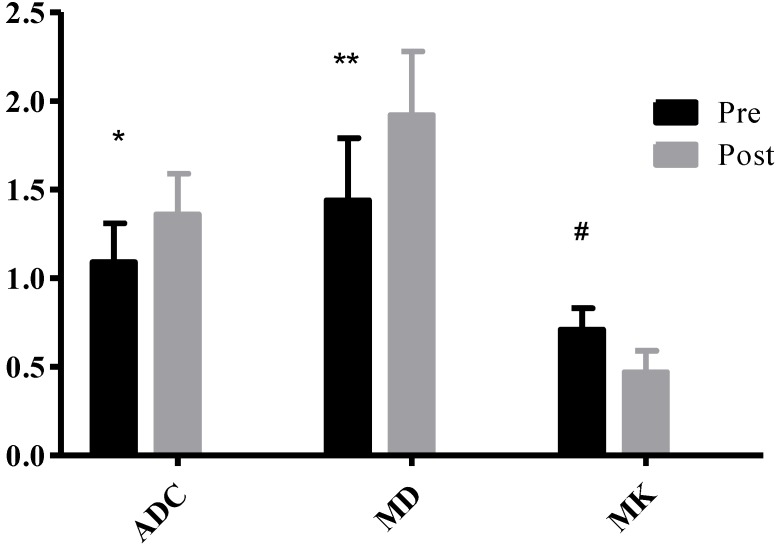
*P<0.05, the Pre versus Post in ADC after TACE; **P<0.05, the Pre versus Post in MD after TACE; ^#^*P*<0.05,the Pre versus Post in MK after TACE.

**Figure 7:**
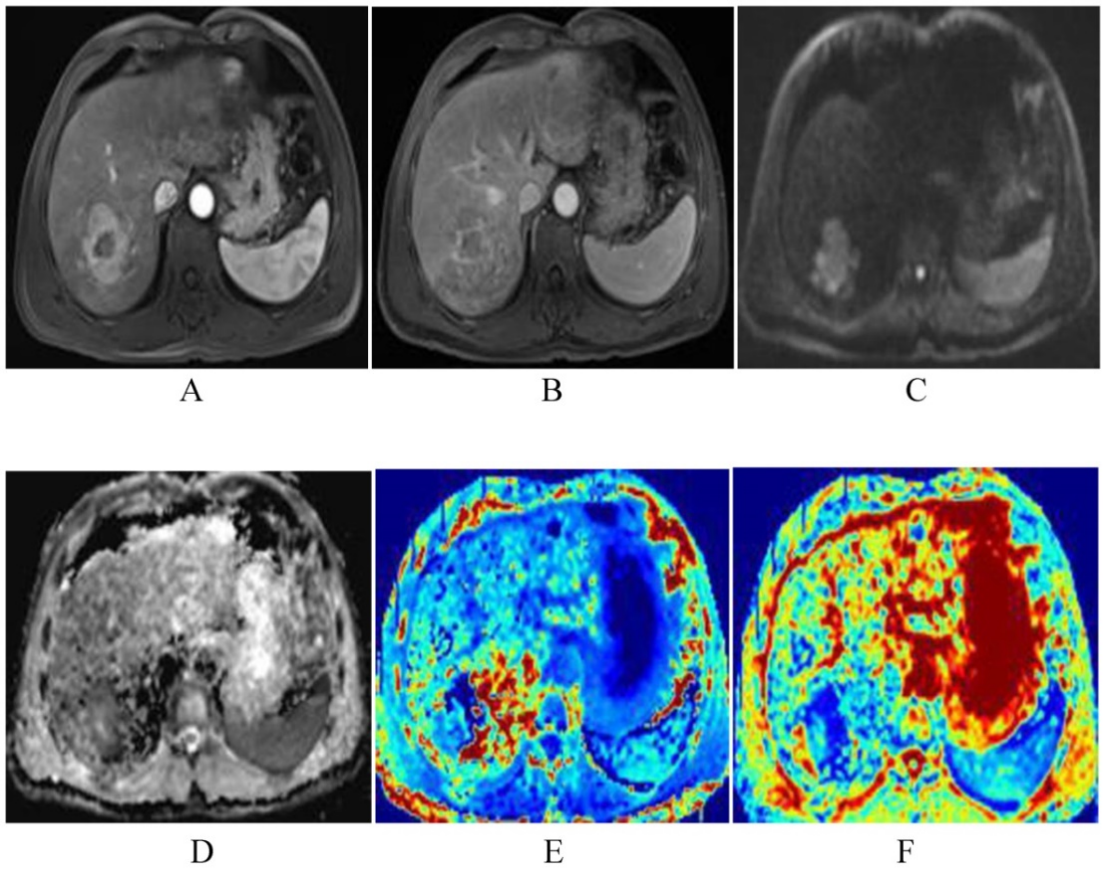
The patient was a 51-year-old man with HCC before TACE. The lesion of the right liver presents significant enhancement of heterogeneity in arterial phase, and decrease enhancement in venous phase, and shows as high DWI signals (A-C). The lesion shows low-signal-intensity in ADC and MD map, higher signal intensity compared with that of liver parenchyma in kurtosis map. The ADC, MD and MK values were 0.89×10^-3^mm^2^/s, 1.45×10^-3^mm^2^/s, 0.85, respectively (D-F).

**Figure 8:**
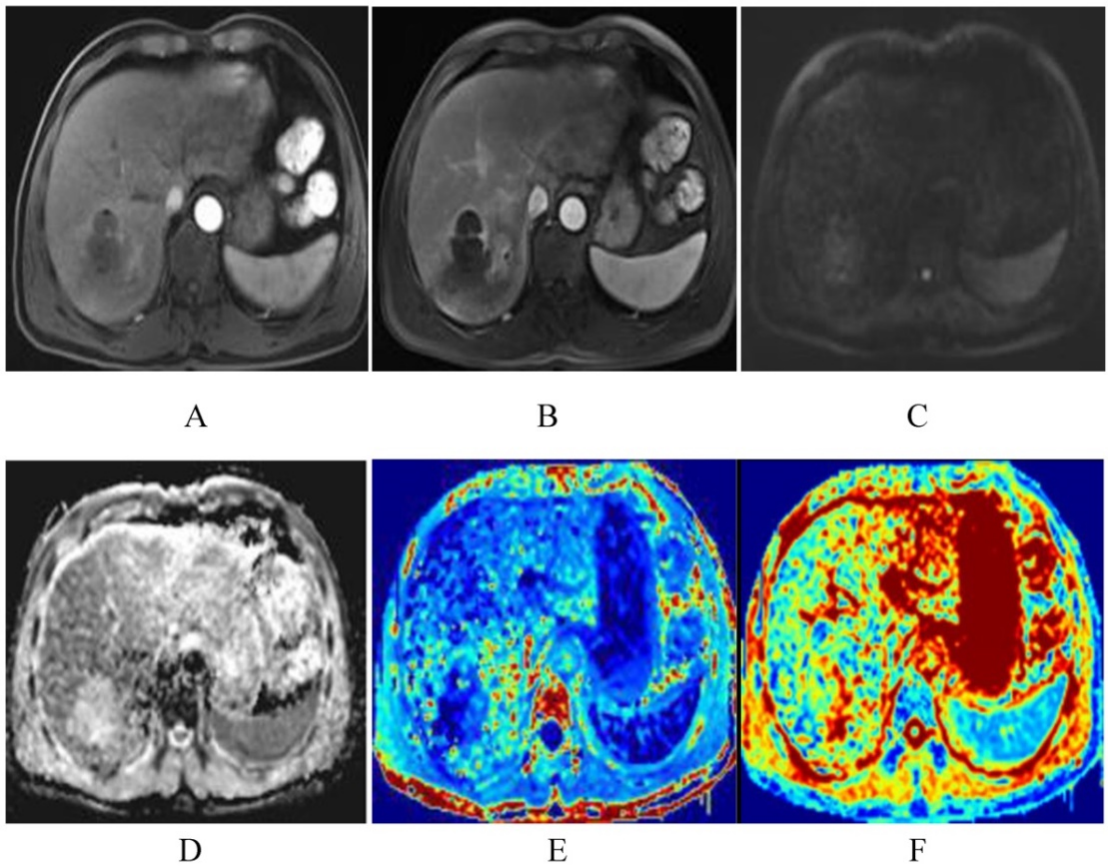
The same patient with Fig. 2 after TACE. The degree of lesion enhancement is significantly reduced and DWI signal is decreased (A-C). ADC and MD map after TACE show higher signal intensity than the residual tumor. The values for the lesion are 1.55×10^-3^mm^2^/s and 2.15×10^-3^mm^2^/s respectively (D-E). Fig. F is the kurtosis map, showing lower signal intensity than the residual tumor. The value for the lesion is 0.45.

Table 2 shows that after TACE, the MK, MD, and ADC values of normal hepatic parenchyma showed no evident changes, compared with the preoperative values (*P*>0.05). In the progressing groups, the MK, MD, and ADC values of tumor remained similar before and one month after TACE (*P*>0.05) (Fig. 5). According to Fig. 6, the MK values of tumor among non-progressing patients decreased one month after TACE (0.47±0.12) relative to the preoperative values (0.71±0.12). A significant difference was observed between the two groups (*P*<0.05). In the non-progressing groups, the MD and ADC values of tumor after TACE (1.92±0.36×10^-3^mm^2^/s, 1.36±0.23× 10^-3^mm^2^/s) became higher than their preoperative values (1.44±0.35×10^-3^mm^2^/s, 1.09±0.22×10^-3^mm^2^/s) (*P*<0.05) (Fig. 7-8).

**Figure 9:**
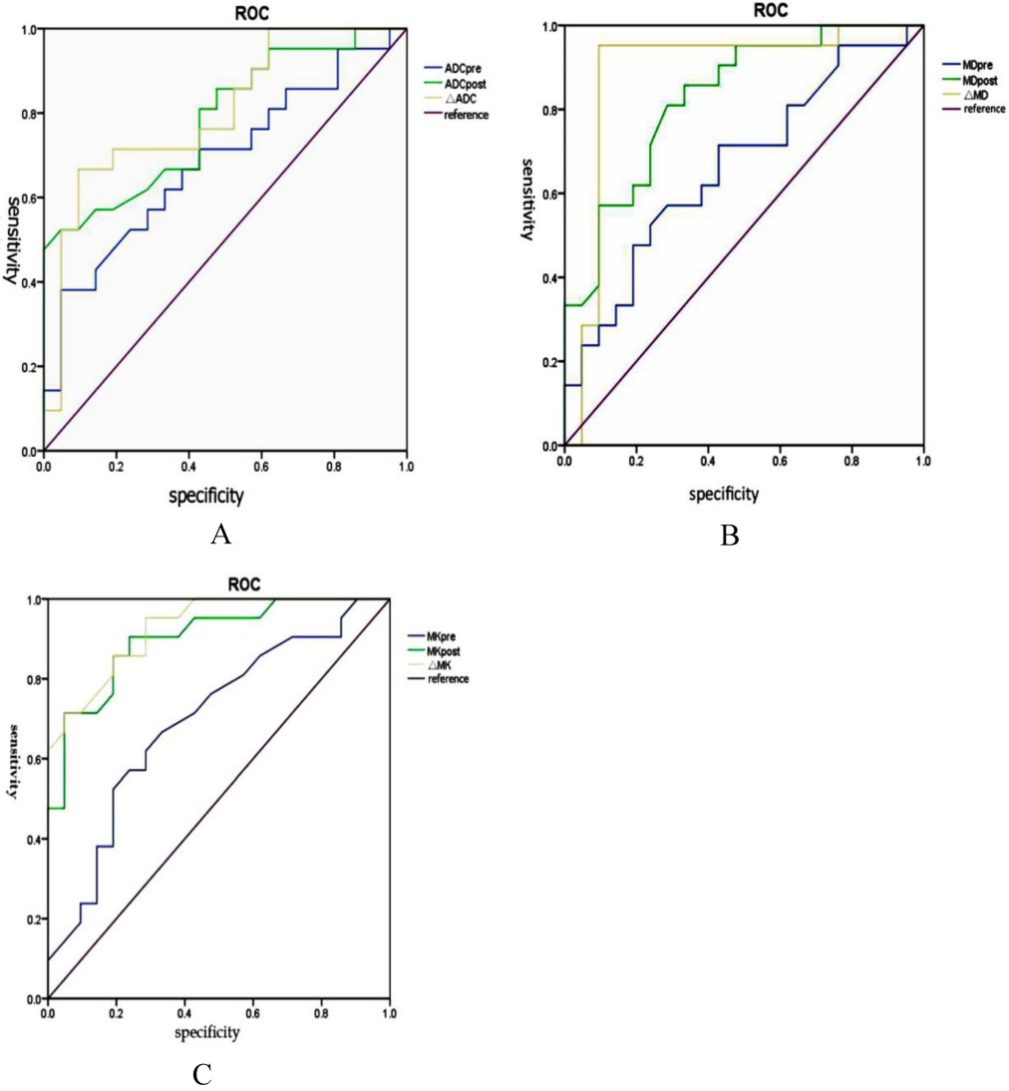
The sensitivity, specificity and AUC of the ROC curve for the assessment of HCC progress after TACE by ADC, MD and MK (A-C).

The sensitivity, specificity and AUC of the ROC curve for the assessment of HCC progress after TACE by using MK (85.2%, 97.5%, and 0.95, respectively) were greater (*P*<0.001) than by ADC (78.6%, 66.5%, and 0.75, respectively). The sensitivity, specificity, and AUC of the ROC curve for the assessment of HCC progress after TACE were greater (*P*<0.05) by MK (85.2%, 97.5%, and 0.95, respectively) than by MD (76.2%, 64.3%, and 0.71, respectively) (Fig. 9). (Table 3. represents the parameters in HCC before and after TACE between non-progressing groups versus progressing groups).

## Discussion

Study showed that complex biological behavior of HCC means high recurrence rate 21. Because of the higher incidence and recurrence rate of HCC, selecting a suitable treatment and prognostic evaluation method was very important. TACE provides a new therapy option for patients with advanced liver cancer, which significantly improved the survival rate of patients with HCC 22. The TACE procedure causes tumor cell apoptosis, necrosis, cell membrane rupture, and nuclear dissolution and thus changes the tissue structure. Necrotic HCC tissues lose their cellularity, usually developing coagulation necrosis, and contain the fewest diffusion barriers. Meanwhile, DKI can evaluate the non-Gaussian distribution of water molecules *in vivo*, reflecting the differences in structures and functions of local tissues and cells. So, DKI can assess the HCC response to the effects of TACE treatment to some extent.

The parameter values of DKI were calculated under its ultra-high b value. B value is diffusion weighted degree (diffusion sensitivity coefficient), which is greatly affected by perfusion. According to the imaging theory of DWI, ADC value, measured at a relatively higher b value, was more sensitive to the detection of the diffusion motion of water molecules. Compared with the conventional DWI sequences, DKI need to set at least three different b values and select a ultra-high b value without affecting the image signal-to-noise ratio to fit the non-gaussian computing model. In the study of brain DKI, the ultra-high b value could be set to 2000~3000s/mm^2^ 23. Recently, the research of DKI technology applied to abdominal shown that when the ultra-high b value is set in the area between 1500 and 2000s/mm^2^, the non-gaussian motion will be well reflected 24-25. In this study, four b values (0, 800, 1,500, 2,000 mm^2^/s) of DKI were selected, respectively. However, in the application of abdomen, DKI should avoid setting too much or too large b values to reduce scanning time, energy consumption and the generation of artifacts 26.

DKI technique was described tissue water molecule motion by analyzing MK and MD 27. ADC also can reflect the degree of diffusion of water molecules by quantification of the diffusion of water molecules with Gaussian distribution. Compared with ADC, MK was more sensitive to the detection of carcinogenic adenoids in the benign surrounding area 28. MK may be a more meaningful indicator for the complexity of the organizational structure compared with MD 29. The study on clear cell renal cell carcinoma shown that MK and MD could distinguish the normal renal parenchyma from clear cell renal cell carcinoma, moreover, MK displays a better performance than MD 16. Moreover, the MK value was significant for evaluating the breast lesion 15. The benign breast lesions had a tendency of significantly lower MK and relatively higher MD values than the malignant tumors. All the above research results are consistent with our study.

The study shows that compared with the preoperative values after TACE, the MK, MD, and ADC values of normal hepatic parenchyma showed no evident changes. This indicated that TACE has little effect on normal liver parenchyma. In addition, on postoperative evaluation of the progressing groups and the non-progressing groups, the result showed that compared with the progressing groups, the MK values were lower, while the values of MD and ADC were greater in the non-progressing groups. This finding can be explained by the necrosis of tumor tissue results in a series of numerical changes. Meanwhile, the changes of each parameter value before and after operation (∆MK, ∆MD, and ∆ADC) were significantly larger in the non-progressing groups than those in the progressing groups. This finding indicated that in the non-progressing groups, TACE method plays an important role in inhibiting tumor growth. In our study, compared with progressing groups, decreased MK and increased ADC and MD were statistically significant in HCC tissues after TACE in the non-progressing groups. However, in comparison, the sensitivity for detection of HCC cell biological characteristics was significantly greater with MK than with ADC and MD. This result was consistent with the previous study 30. This finding demonstrated that a lower MK value indicates evidence of necrosis and reflects the changes of the biological characteristics of tumor cells after TACE. In general, the normal liver parenchyma shows homogeneous density and contains many barriers for diffusion, such as liver cells, fibrous septa, and sinusoids. After TACE, necrotic HCCs lose their cellularity, usually developing coagulation necrosis, and contain the fewest diffusion barriers. Thus, the non-Gaussian movement of the tumor tissue water molecules moved freely. Meanwhile, obvious proliferation of tumor cells and the formation of neovascularization in the progressing group were found. These changes lead to decreased MK in non-progressing groups. Therefore, the differences in MK values observed in our study reflected the differences in tissue microstructural complexity between the progressing and non-progressing groups. The change of MK values before and after TACE could thus be used to estimate the degree of tumor necrosis and to further evaluate the effect of interventional therapy.

## Conclusion

In conclusion, DKI is a preference diffusion technique, which can provide valuable information on the necrosis of HCC after TACE. MK is more advantageous in the assessment of HCC progress after TACE than by using ADC and MD. Thus, DKI shows great promise for assessing the therapeutic response of TACE in HCC.

## Figures and Tables

**Table 1:** Clinical characteristic of the patients and HCCs

	Numbers
**Liver cirrhosis (n)**	29
**Acute or chronic hepatitis (n)**	32
**Child-Pugh A/B /C(n)**	23/14/6
**Serum AFP levels, ng/ml, mean±SD**	73.3±281.2
**Albumin, g/L, mean±SD**	45.2±8.3
**Bilirubin, μmol/L, mean±SD**	20.9±10.1
**Platelets, ×1000/ml**	160.3±60.2
**Completed envelope(n)**	40
**Metastasis(n)**	
Local metastasis	14
Distant metastasis	9
**Vascular invasion(n)**	8
**HCC median size, cm**	2.3±0.6
**Number of tumors one/two/three**	30/10/3
**Edmondson grade 1 or 2(n)**	26
**Edmondson grade 3 or 4(n)**	17

*Available in 43 patients.

**Table 2:** The parameters of normal liver parenchyma before and after TACE.

Liver parenchyma	Non-progressing groups				Progressing groups		
	Pre	Post	*P* value		Pre	Post	*P* value
**ADC(×10^-3^mm^2^/s)**	1.32	1.28	>0.05		1.29	1.28	>0.05
**MD(×10^-3^mm^2^/s)**	1.67	1.61	>0.05		1.64	1.59	>0.05
**MK**	0.98	1.02	>0.05		1.01	1.06	>0.05

**Table 3:** The parameters in HCC before and after TACE between non-progressing groups versus progressing groups

		Non-progressing groups	Progress groups	*P* value
**ADC(×10^-3^mm^2^/s)**	Pre	1.09±0.20	0.97±0.17	0.040
	Post	1.36±0.23	1.10±0.23	0.002
**MD(×10^-3^mm^2^/s)**	Pre	1.44±0.35	1.24±0.28	0.066
	Post	1.92±0.36	1.44±0.32	0.000
**MK**	Pre	0.71±0.12	0.81±0.11	0.029
	Post	0.47±0.12	0.72±0.14	0.000
**∆ADC(×10^-3^mm^2^/s)**		0.27±0.13	0.19±0.10	0.001
**∆MD(×10^-3^mm^2^/s)**		0.48±0.10	0.21±0.13	0.000
**∆MK**		0.25±0.07	0.09±0.08	0.000
